# Management Options for Linear Immunoglobulin A (IgA) Bullous Dermatosis: A Literature Review

**DOI:** 10.7759/cureus.36481

**Published:** 2023-03-21

**Authors:** Madiha Khan, Lily Park, Stanley Skopit

**Affiliations:** 1 Department of Dermatology, New York Institute of Technology College of Osteopathic Medicine, Old Westbury, USA; 2 Department of Dermatology, Larkin Community Hospital, Miami, USA; 3 Department of Dermatology, Dr. Kiran C. Patel College of Osteopathic Medicine, Nova Southeastern University (NSU) Florida, Fort Lauderdale, USA

**Keywords:** chronic bullous disease of childhood, labd, linear iga bullous dermatosis, autoimmune bullous dermatoses, linear iga dermatosis

## Abstract

Linear immunoglobulin A (IgA) bullous dermatosis (LABD) is an autoimmune condition with various triggers. Because of the lack of randomized controlled trials on LABD treatment, management options are mostly anecdotal. This paper provides a comprehensive review of treatment options from a literature review of reported treatments to arm clinicians with a guideline for the management of LABD in both pediatric and adult patients as well as those recalcitrant to first-line therapy (dapsone and steroids). We additionally illustrate an algorithm to use for the management of LABD to aid clinicians when faced with unique patient circumstances.

## Introduction and background

Linear immunoglobulin A (IgA) bullous dermatosis (LABD) is an autoimmune blistering disease notable for subepidermal blisters and linear IgA basement membrane antibody deposition [1]. It was originally thought to be a subtype of dermatitis herpetiformis or bullous pemphigoid but is currently regarded as a separate pathology [2]. There is often no specific trigger identified in LABD. However, there are well-known etiologies such as medications, skin trauma, malignancy (chronic lymphoproliferative lymphoma and renal cell carcinoma), and gastrointestinal (GI) disorders [1]. For drug-induced LABD, vancomycin is one of the most well-known causes although numerous other medications can be triggers. In many drug-induced cases, the disease course of LABD is acute and resolves if the culprit medication is discontinued swiftly.

The incidence of LABD is 0.5 per million in western Europe, making it a rare disease [1]. It is observed more commonly in other countries, including China, Southeast Asia, and Africa, due to a more frequent number of childhood diseases [1]. LABD demonstrates a bimodal distribution of onset: early childhood and older than 60 years of age [1]. When present in the pediatric population, the term chronic bullous disease of childhood (CBDC) is used. The incidence of this disease is slightly higher among women; however, it is relatively equal among the sexes [1].

The pathophysiology of LABD has been postulated to be due to IgA antibodies targeting BP180/collagen XVII, which produces infiltration of neutrophils, resulting in vesicle formation on the basement membrane [1]. Other antigens that have been reported include BP230, LAD285, collagen VII, and other dermal antigens which have not yet been elucidated [1]. The mechanism of drug-induced LABD is not yet known. Possibly, the drugs act as haptens, which induce an autoimmune response, creating autoantigens via complexes with cutaneous proteins. Vancomycin-induced LABD specifically has been studied to exhibit IgA autoreactivity against the NC1 domain of type VII collagen.

Although LABD can affect both the pediatric population and adults, the clinical presentation varies between the two even with the same underlying pathology [1]. In children, the average age of onset ranges from four to five years. Acutely blistered skin with possible associated mild pruritus or severe burning is the typical presentation in a child [1]. This acute presentation is typically more severe than subsequent episodes. Areas that are typically affected include the face, perineal area, lower abdomen, vulva, thighs, and buttocks [1]. Of note, the hands and feet may also become involved as the lesions advance. The typical appearance of the lesions is a urticated plaque with an annular or polycyclic pattern. The presence of blisters surrounding the perimeter creates the *string-of-pearls* sign [1]. Enlargement or hemorrhage of these surrounding blisters can occur as well. Ulceration or erosion of the mucosa, as well as nasal congestion and conjunctivitis, is commonly present.

In an adult, the average age of onset is between 60 and 65 years, and either rapid or slow onset may occur. Similarly, in children, symptoms of pruritus or burning may be present. Common locations of the lesions include the trunk, face, scalp, and extremities. Less commonly, vaginal or genital manifestations can be present as well [1]. In both children and adults, mucosal involvement is observed in 60% to 80% of the population. Urticated plaques and papules with vesicles and blisters characterize these lesions. The vesicles or blisters can emerge from normal skin or overly the area of urticated plaques [1]. The *string-of-pearls* sign and annular appearance are not as prevalent in adults as they are in children [1]. Nasal congestion and ocular manifestations are also common in adults. Some patients may note a hoarse voice, which hints that the pharynx may be affected.

The diagnosis of LABD requires a clinicopathologic correlation for a definitive diagnosis. Histologic features are not specific to this disease [1]. The features observed include subepidermal blisters and nonspecific cellular infiltrate [1]. The overwhelming presence of eosinophils may be observed, and thus, histology may be mistaken as bullous pemphigoid [1]. There may also be a neutrophil presence with dermal papillary microabscesses easily mistaken as dermatitis herpetiformis pathology [1]. Nonspecific histologic features make it necessary to obtain a direct immunofluorescence (DIF) sample to delineate the pathology. Skin such as the back that is not visually marred by lesions should be the site of DIF sampling [1]. IgA arranged linearly (rarely a granular linear pattern) along the basement membrane zone is observed on DIF [1].

There are currently no guidelines on the management of LABD or CBDC, although numerous treatment options have been utilized and reported in the literature, mainly in the form of case reports and retrospective studies. This paper provides a comprehensive review of treatment options from a literature review of reported treatments to arm clinicians with a guideline for the management of LABD in both pediatric and adult patients as well as those recalcitrant to first-line therapy (dapsone and steroids). We additionally illustrate an algorithm to use for the management of LABD to aid clinicians when faced with unique patient circumstances.

## Review

Methods

The search terms "linear IgA bullous dermatosis treatment," "LABD treatment," "chronic bullous disease of childhood treatment," and "CBDC treatment" were queried using the PubMed database for studies from 1980 to the present. After noting the various treatment options that were reported in the literature, the aforementioned search terms were queried in conjunction with the individual drug names to determine the number of studies utilizing that particular treatment option. Inclusion criteria included the treatment of LABD or CBDC with medications other than dapsone or corticosteroids alone. Exclusion criteria consisted of studies lacking details about patient treatment and course.

Results

Our search yielded 930 studies. After applying inclusion and exclusion criteria as well as removing duplicates, 69 studies were included in this review. Forty-eight were case reports, 12 were case series, eight were retrospective studies, and one was a cross-sectional study.

First-line* *treatment

Because many cases of LABD are drug-induced, a comprehensive history and physical exam are the first step in the diagnosis. If a patient has recently started a drug, especially one reported to cause LABD, immediate discontinuation of the drug should be considered the prime treatment. In the event the drug discontinuation is unsuccessful or the patient has severe LABD, dapsone in the range of 50-150 mg/day for adults is the first-line treatment [1,2]. Lower doses (0.5-2 mg/kg/day) are utilized for children [3]. It is imperative to first check a glucose-6-phosphate dehydrogenase (G6PD) level before initiating dapsone as G6PD deficiency can cause life-threatening hemolysis. Other notable adverse effects of dapsone include dose-related methemoglobinemia, cholestatic jaundice, bone marrow suppression, hypersensitivity reactions, and motor peripheral neuropathy [1]. In theory, cimetidine can be utilized along with dapsone as it increases tolerability and decreases hematotoxicity via inhibition of the hydroxylamine metabolites. However, cimetidine is rarely prescribed with dapsone in practical settings [4].

Other first-line or adjunctive treatments

If the patient has G6PD deficiency or sulfa allergy preventing dapsone use, oral corticosteroids alone may be utilized, even in drug-induced cases. For patients with mild LABD, topical steroids with high potency such as clobetasol propionate 0.05% may be considered as the sole treatment option as well, with the possible addition of dapsone if the disease remains uncleared [5]. Intermittent drug holidays from corticosteroids are implemented to avoid skin atrophy, striae, or telangiectasias of the skin. Additionally, in patients without G6PD deficiency or sulfa allergy with LABD incompletely controlled with dapsone, oral corticosteroids may be added as an adjunctive treatment. Intravenous (IV) or intramuscular (IM) corticosteroid injections may also be utilized depending on the specific patient circumstance (i.e., inability to take medication by mouth) and disease severity (i.e., a hospitalized patient with severe LABD requiring IV or IM steroids). Prednisolone dose of 0.5-1 mg/kg/day can be utilized in the pediatric population, while a dose of up to 1 mg/kg/day in adults may be tried [5]. Monitoring of adverse effects such as Cushing's syndrome should be implemented if steroids are used for a prolonged period [5]. Other adverse effects include infections, osteoporosis, cataract, elevation in blood sugar, hyperlipidemia, and psychiatric changes.

Sulfonamides such as sulfapyridine or sulfamethoxypyridazine are alternative treatments in the event dapsone with or without oral corticosteroids is ineffective, although they may not be readily available and are inferior to dapsone. Sulfonamides can also be used in conjunction with dapsone and topical/oral corticosteroids for LABD resistant to dapsone alone. The reported dose of sulfapyridine in adults ranges from 1,000 to 1,500 mg/day, while the range for children has been reported as 15-60 mg/kg/day [3]. Sulfonamides have similar adverse effects as dapsone; hypersensitivity to dapsone does not hinder the use of these medications, although it is best to avoid its use due to its potential for overlapping hypersensitivity [6].

**Figure 1:**
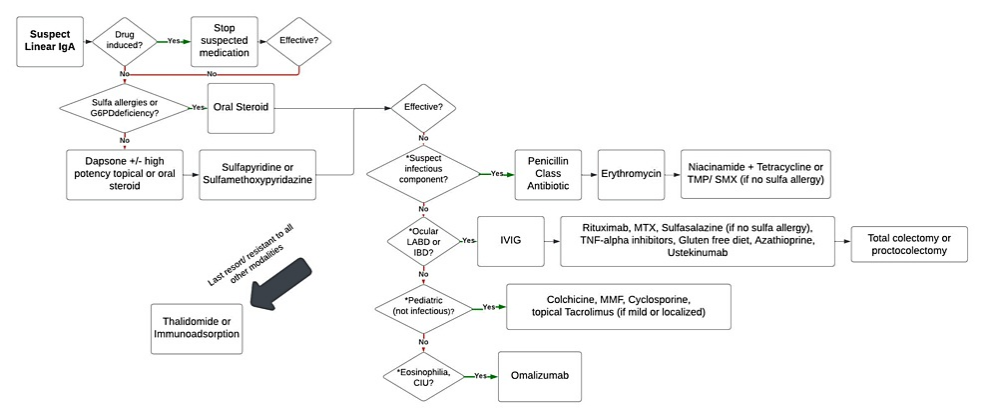
Algorithm for managing LABD. *Lack of these conditions does not preclude the use of the following treatments for recalcitrant LABD. Take specific risks versus benefits of each treatment into account for each patient. Figure credits: All the authors of this study. CIU, chronic idiopathic urticaria; G6PD deficiency, glucose-6-phosphate dehydrogenase deficiency; IBD, inflammatory bowel disease; IVIG, intravenous immunoglobulin; LABD, linear IgA bullous dermatosis; MMF, mycophenolate mofetil; MTX, methotrexate; TNF-alpha inhibitors, tumor necrosis factor-alpha inhibitors; TMP/SMX, trimethoprim/sulfamethoxazole

Numerous alternative treatments for LABD have been reported. Because substantial randomized controlled trials are lacking in the literature for LABD treatment, management options are based largely on anecdotal evidence. We describe below additional management options for LABD in order of greatest to least evidence based on the quantity of supporting literature and treatment success (Table 1). We additionally present an algorithm for the management of LABD (Figure 1).

**Table 1:** Management options of LABD listed from most evidence to least based on the quantity of supporting literature and treatment success; reported utilities, contraindications, and notable adverse effects are also listed. CHF, congestive heart failure; CI, contraindication; GI, gastrointestinal; hx, history; HTN, hypertension; IBD, inflammatory bowel disease; IVIG, intravenous immunoglobulin; LABD, linear IgA bullous disease; MG, myasthenia gravis; SJS/TEN, Stevens-Johnson syndrome/toxic epidermal necrolysis; SIADH, syndrome of inappropriate antidiuretic hormone; sx, symptoms; TB, tuberculosis; tx, treatment; URI, upper respiratory infection

Drug	Utility	CI (other than history of allergy/hypersensitivity)	Potential AE
Penicillin antibiotics (amoxicillin-clavulanate, flucloxacillin, dicloxacillin, cloxacillin, oxacillin, penicillin A)	Pediatrics or adults with possible infectious etiology	hx of SJS/TEN, liver abnormalities (if using flucloxacillin)	Rash and cholestatic hepatitis (flucloxacillin)
IVIG	Ocular LABD and IBD	Severe renal failure	Chills and headache
Colchicine	Pediatrics or adults	Renal insufficiency	GI sx
Rituximab	IBD	Severe CHF and pregnancy	Fever, chills, body aches, infections, and SIADH
Erythromycin	Pediatrics or adults	Hx of MG or QT prolongation	Diarrhea and QT interval prolongation
Mycophenolate mofetil	Pediatrics or adults and mucosal surface involvement	Severe liver or renal abnormalities and pregnancy	GI sx, hepatotoxicity, and nephrotoxicity
Nicotinamide/Niacinamide + Tetracycline	Elderly patients or renal failure	Children <8 years unless using without tetracycline, pregnancy	GI sx
Total colectomy/ proctocolectomy	IBD	Poor surgical candidate	Complications from surgery
Methotrexate	IBD or concomitant psoriasis	Liver abnormalities and pregnancy	Hepatotoxicity and pulmonary fibrosis
Cyclosporine	Pediatrics or adults	Malignancy, uncontrolled HTN, renal insufficiency, and pregnancy	Hirsutism, gingival hyperplasia, and HTN
TNF-alpha inhibitors (certolizumab, etanercept, and infliximab)	Pregnancy and IBD	Latent TB	Injection site reaction and URI
Omalizumab	LABD with eosinophilia or elevated IgE and concomitant chronic idiopathic urticaria	-	US boxed warning of anaphylaxis (0.1%-0.2% incidence)
Sulfasalazine	IBD	Sulfa allergies	GI sx
Trimethoprim/Sulfamethoxazole	Pediatrics or adults	Sulfa allergy, first trimester pregnancy, and hx SJS/TEN	Myelosuppression
Gluten-free diet	Mild LABD	-	-
Tacrolimus (topical)	Patients with the preference for topical agents	-	Skin stinging or burning
Azathioprine	IBD and pregnancy	Severe neutropenia	GI sx, hair loss, and rash
Ustekinumab	IBD and mucosal LABD	-	Nasal congestion, URI, and injection site reaction
Thalidomide	Last resort tx	Pregnancy	Neuropathy
Immunoadsorption	Quick remission induction or last resort tx	Expensive and needs special equipment	Dizziness and nausea

Additional treatment considerations

Penicillin Class

Amoxicillin-clavulanate at a dose of 1 g/day has induced remission of CBDC and has been used in controlling CBDC in a child-resistant with dapsone and oral steroids [7,8]. The treatment duration has varied from seven days to months, making the length of treatment patient-dependent. Of note, amoxicillin-clavulanate has also been implicated as the inciting factor in cases of CBDC [9].

Cloxacillin [10], dicloxacillin [11,12], flucloxacillin [13,14], oxacillin [15,16], and penicillin A [17] have also been utilized in literature for the treatment of LABD. The outcome of using these antibiotics is variable. There have been reports of early treatment with flucloxacillin within one month of the onset of lesions, leading to complete remission. Side effects of flucloxacillin include cholestatic hepatitis, aplastic anemia, hemolytic anemia, agranulocytosis, and acute interstitial nephritis.

IV Immunoglobulin 

Intravenous immunoglobulin (IVIG) has been utilized in cases recalcitrant to standard therapies. Khan et al. [18] reported a case of a 50-year-old woman with renal failure who developed complications from standard LABD treatment. After failing dapsone, tetracycline, nicotinamide, and systemic antibiotics, the patient received a total of 4 g/kg of IVIG per month with remission after six months. Increasing the infusion interval resulted in recurrence, requiring maintenance infusion every eight weeks.

IVIG has additionally been used in other cases with similar outcomes and may be a possible adjunctive treatment for patients with comorbidities such as inflammatory bowel disease (IBD) or LABD limited to the eye [19-24].

Colchicine

Colchicine is an anti-inflammatory drug that inhibits neutrophil and monocyte chemotaxis as well as leukocyte adhesion. Seven case reports have been described utilizing colchicine [25-31]. One study by Ang and Tay [25] described the case of a five-year-old girl who developed CBDC one month after the resolution of hand-foot-mouth disease. After discontinuation of dapsone and prednisone due to hemolytic anemia, colchicine 0.5 mg twice daily was initiated with remission within two weeks. This medication may be better suited for the pediatric population as it is well tolerated. Adverse effects typically occur at high doses and include nausea, vomiting, diarrhea, or abdominal pain.

Rituximab

Rituximab is an anti-CD20 monoclonal antibody and has recently been reported as an effective treatment for recalcitrant LABD [32-38]. Pinard et al. [32] reported two patients (males, aged 35 and 30 years) with LABD, who were successfully treated with rituximab. The 35-year-old male had a history of sclerosing cholangitis and Crohn’s disease and was initially started on dapsone with topical corticosteroids without response. Cyclosporine was also tried; however, still, no disease control was achieved. Subsequently, he was given two infusions of 1 g of rituximab two weeks apart with mild improvement. A second 1 g infusion was given six months after the first infusion. He was placed on 3 g/day mycophenolate mofetil (MMF) for maintenance. The lesions were resolved completely nine months after the second rituximab infusion with a recurrence, which resolved with two more 1 g infusions of rituximab. MMF was subsequently decreased to 500 mg/day without a recurrence.

The second patient, aged 30 years, had severe LABD for 20 years with oral mucosal involvement. He had previously been treated on dapsone; however, the relapse occurred when it was discontinued. Retreatment with dapsone was not effective. He was started on low-dose prednisone 10 years before evaluation. He was also currently taking MMF 1 g/day for eight years. Dapsone 200 mg/day was again tried along with 200 mg/day doxycycline with the continuation of the MMF and prednisone without complete resolution. Therefore, two 1 g infusions of rituximab were given two weeks apart. Due to incomplete resolution, two more 1 g infusions were administered two weeks apart, eight months after the initial infusion. Remission was maintained for one year after the last rituximab infusion, allowing discontinuation of doxycycline and decrease of dapsone and MMF doses to 150 and 750 mg/day, respectively.

Nedosekin et al. [33] additionally described the use of rituximab in a 43-year-old patient with a history of end-stage renal disease presenting with resistant linear IgA/IgG bullous dermatosis. Topical triamcinolone and two weeks of 60 mg oral prednisone did not provide resolution. Rituximab 375 mg/m^2^/week was initiated for four weeks along with 500 mg twice a day of MMF and an increased prednisone dose to 80 mg. The patient was not placed on dapsone secondary to anemia. The patient had a resolution after four rituximab infusions, allowing the tapering of prednisone. Recurrence occurred after nine months; however, they were resolved with two more rituximab infusions. Adverse effects of rituximab include infections, syndrome of inappropriate antidiuretic hormone, drug fever, and tachycardia. For patients with multiple complex comorbidities such as IBD and severe disease, rituximab can provide a lasting resolution.

Erythromycin

Erythromycin is a macrolide antibiotic that has been utilized in many cases for the treatment of LABD in both the adult and pediatric populations [8,10,15,39-41]. Powell et al. [39] reported a six-year-old female with CBDC responsive to 250 mg erythromycin three times daily along with topical steroids. The patient’s blistering resolved within two weeks; however, it recurred upon discontinuation of erythromycin, requiring repeat doses to abate the lesions. Cooper et al. [40] described a case of a 52-year-old male who had chronic LABD treated intermittently with dapsone; however, a subsequent flare was not adequately controlled even with 300 mg of dapsone. He was started on 500 mg clarithromycin twice a day (BID) due to another minor infection, which surprisingly decreased the blistering. Unfortunately, the patient developed urticarial lesions, prompting the discontinuation of clarithromycin. Erythromycin 500 mg four times a day (QID) was tried for 10 days, which resolved his lesions without the development of urticaria. The second patient reported by Cooper et al. was a five-year-old (female) who failed topical steroid therapy; however, the patient did respond to 250 mg erythromycin three times daily (TID). She experienced flares upon discontinuation of erythromycin, prompting continuous maintenance treatment with erythromycin 250 mg daily remaining well controlled after 24 months of treatment. Erythromycin is also advantageous due to its safety profile with limited adverse effects with no necessary laboratory monitoring.

Mycophenolate Mofetil

MMF is a corticosteroid-sparing agent that binds inosine monophosphate dehydrogenase and prevents the biosynthesis of purines. As lymphocytes utilize the de novo pathway for purine synthesis, blocking the T and B lymphocyte response leads to reduced antibody production and resolution of the autoimmune blistering disease. Other than for prevention of solid organ transplant rejection, MMF has been used to treat autoimmune diseases such as psoriasis, bullous pemphigoid, pemphigus vulgaris, and CBDC or LABD patients [42-47]. Reported dosages used in the treatment of LABD are 2 g/day for 5 to 14 weeks, while in CBDC, the dosage has been reported in the range of 155 to 310 mg/m^2^ daily for eight months [42,43]. Maintenance MMF dose has been reported at 250 mg to 1 g daily, with complete resolution after 9-32 months [44,45]. Although generally well tolerated, MMF can rarely cause gastrointestinal symptoms, hepatotoxicity, nephrotoxicity, anemia, neutropenia, and leukopenia, which can lead to susceptibility to infections. Headaches and disturbances in sleep have also been reported.

Nicotinamide/Niacinamide + Tetracycline

The combination of tetracycline and nicotinamide (niacinamide) has been utilized as well [48-52]. Dosages range from 1.5 to 2 g of tetracycline with 900 mg to 1.5 g of niacinamide daily, with resolution as early as three weeks, although treatment periods have been reported up to two years [48,49]. Other pathologies treated with either nicotinamide by itself or combined with tetracycline include dermatitis herpetiformis, necrobiosis lipoidica diabeticorum, generalized granuloma annulare, polymorphous light eruption, and pellagra. Tetracycline and nicotinamide may aid in the treatment of autoimmune blistering conditions by proposed inhibition of neutrophil and/or eosinophil chemotaxis, histamine release, phosphodiesterase, and transformation of lymphocytes [48]. Interestingly, nicotinamide alone has also been utilized in one case of CBDC at a dose of 300 mg/day, which provided resolution in seven days, making nicotinamide a possible option for the pediatric population [52].

The combination of tetracycline and nicotinamide may be a viable option for older patients to spare the use of steroids, which come with the risk of numerous adverse effects. This combination also possesses lower toxicity as compared to corticosteroids and immunosuppressant agents. The main adverse effect of tetracycline/nicotinamide includes gastrointestinal disturbance. Other less common side effects include flushing, pruritus, headache, vomiting, and hepatotoxicity. Notably, tetracyclines are generally avoided in young children and pregnant women due to their possible side effects on the dentition.

Total Colectomy/Proctocolectomy

There have been five case reports of people with LABD associated with IBD who were treated with total colectomy or proctocolectomy due to insufficient response to adalimumab, mesalazine, prednisone, or dapsone for months [53-57]. Complete resolution was achieved with no need for maintenance medication or relapse after years. Due to remission after colectomy, the colon or rectum may be the source of antigenicity. Proctocolectomy illustrated a better incidence of lasting LABD resolution as compared to colectomy.

Methotrexate

Methotrexate (MTX) is a dihydrofolate reductase inhibitor used in autoimmune conditions as well as for the treatment of certain malignancies. Four case reports have been reported in the literature of MTX use in LABD [35,58-60]. Yetto and Burns [58] described a case of a mild LABD eruption associated with ulcerative colitis [58]. Mesalamine along with 22.5 mg of MTX weekly resolved his lesions. Dosages in the range of 10-15 mg/week have also been successful in treating LABD. Therefore, MTX may be a treatment option in LABD patients, especially with associated IBD. Major adverse effects to be aware of include hepatotoxicity, hematologic abnormalities, and pulmonary fibrosis from prolonged use.

Cyclosporine

Cyclosporine is a calcineurin inhibitor that blocks the synthesis of interleukins (IL) such as IL-2 necessary for T-lymphocyte activation and differentiation. It has been used adjunctively with dapsone and prednisolone for recalcitrant LABD and CBDC at a dose of 3 mg/kg/day with resolution as early as three weeks, although requiring ongoing treatment for over three months to prevent recurrence [59,61,62]. Major adverse effects of cyclosporine include hepatoxicity and nephrotoxicity. To allow tapering of systemic steroids and dapsone, cyclosporine can be considered as an adjunctive treatment option in both the adult and pediatric populations.

TNF-Alpha Inhibitors

TNF-alpha inhibitors such as certolizumab, etanercept, and infliximab have also been utilized in LABD treatment associated with IBD or for drug-induced LABD with favorable response [63-66]. Dosages for etanercept are typically 50 mg subcutaneously every week. Certolizumab is dosed at 400 mg subcutaneously every four weeks, while infliximab is dosed at 5 mg/kg intravenously on weeks 0, 2, and 6. Adverse effects include injection site reaction and upper respiratory infections.

Omalizumab

Omalizumab is a monoclonal anti-IgE antibody approved for chronic idiopathic urticaria (CIU) and has also been successfully utilized in treatment for LABD patients in a few reports [67-69]. The efficacy of omalizumab alludes to a possible role of IgE in the pathogenesis of LABD. The mechanism of action of omalizumab involves the prevention of the interaction between free circulating IgE and mast cell/basophil receptors, which subsequently release inflammatory cytokines important in eosinophil production [67]. The dosage utilized is typically 300 mg subcutaneously every four weeks. The treatment duration varies but has been reported between six and 12 months, although standard therapy is for a six-month duration [67,68]. Although omalizumab has been used as a standalone therapy in one case, other cases have utilized it alongside standard therapies such as sulfapyridine 1 g daily to optimize resolution [67,68]. No routine bloodwork is required while on omalizumab; before initiating therapy, a blood IgE level should be checked to optimize dosing. For patients with histologic evidence of eosinophils or peripheral blood eosinophilia along with an urticarial component, omalizumab should be considered as a treatment for refractory cases.

Sulfasalazine

Sulfasalazine is an anti-inflammatory medication commonly used to treat IBD [70,71]. Cetkovska et al. [70] reported a 44-year-old woman presenting with linear IgA of the scalp, trunk, and extremities was initially treated with 150 mg of dapsone with a resolution of lesions. However, she developed a hypersensitivity reaction with the development of methemoglobinemia, morbilliform rash, and additional mucosal involvement, which prompted dapsone discontinuation and the addition of oral corticosteroids instead. Due to the nonresolution of the vesicular lesions, 1,500 mg sulfasalazine was started with disease remission on a maintenance regimen of 1,000 mg sulfasalazine with low-dose prednisone. For patients with dapsone hypersensitivity or a lack of access to it, barring any sulfa allergies, sulfasalazine may be a good treatment option.

Trimethoprim/Sulfamethoxazole

Trimethoprim/sulfamethoxazole (TMP/SMX) is an antifolate antibiotic within the sulfa class reported to have immunosuppressant and anti-inflammatory action. Two case reports have described its successful use in LABD and CBDC [72,73]. Dosage ranges from TMP/SMX 160 mg/800 mg twice daily to 320 mg/1600 mg twice daily for adults and 40 mg/200 mg twice daily for the pediatric population. The treatment duration is variable but has been reportedly used for over 10 months. Adverse effects of TMP/SMX include myelosuppression, Stevens-Johnson syndrome, and toxic epidermal necrolysis. Interestingly, there have been some case reports describing TMP/SMX-induced LABD [74,75].

Topical Tacrolimus

Tacrolimus is a calcineurin inhibitor, which suppresses T-cell activation, which, in turn, decreases ILs (IL-2, IL-3, and Il-4), granulocyte colony-stimulating factor, and tumor necrosis factor production. Because the pathogenesis of LABD involves T-cell activation, tacrolimus 0.03% ointment may have a therapeutic role in treatment as it was successfully used in one case of resistant CBDC despite treatment with dapsone and topical corticosteroids [76]. Although no case reports utilizing pimecrolimus have been published, it may also be a potential option as both pimecrolimus and tacrolimus are topical calcineurin inhibitors. Therefore, topical tacrolimus and potentially topical pimecrolimus may be considered for milder localized cases of LABD or CBDC resistant to topical steroids or dapsone.

Azathioprine

Azathioprine is a purine synthesis inhibitor used to prevent transplant rejection and treat IBD or other inflammatory diseases. It has been listed in a retrospective study as an adjunctive treatment for LABD along with prednisone and dapsone with complete resolution in two adult females (aged 51 and 61 years without comorbidities) even after an 18- and 36-month follow-up, respectively [34]. Dosage is variable but ranges from 1 to 3 mg/kg daily. Before starting therapy with azathioprine, the creatinine level must be checked; additionally, the complete blood count and liver function tests should be obtained weekly for four weeks, then every two weeks for eight weeks, and then monthly while on the medication.

Ustekinumab

Ustekinumab is a monoclonal antibody that specifically binds the IL-12/23 p40 subunit. Jimenez et al. [77] reported the case of successful utilization of ustekinumab in an LABD patient with a history of Crohn’s disease and common variable immune deficiency, resistant to other therapies [77]. The dose utilized was 90 mg subcutaneously every eight weeks with successful maintenance of remission at one year. Notably, one case report of ustekinumab-induced LABD in a psoriasis patient has also been reported [78]. Nevertheless, it remains a treatment option if others fail, especially in patients with underlying concomitant autoimmune disease. Adverse effects to monitor include injection site reaction, upper respiratory infection, and headaches. Tuberculosis testing at baseline must be conducted before initiating therapy.

Thalidomide

Thalidomide is an anti-inflammatory medication that has been used for the treatment of malignancies as well as autoimmune conditions such as pemphigus vulgaris and cicatricial pemphigoid. In one case report, thalidomide 3 mg/kg/day was utilized as a treatment for CBDC with a resolution of lesions within one month although the patient later developed peripheral neuropathy in one leg [4]. Thalidomide can be used in select patients who can tolerate the medication and those who are not pregnant or capable of childbearing.

Immunoadsorption

Immunoadsorption (IA) is a modality utilized in immunobullous diseases, specifically IgG-mediated. IA, as compared to plasmapheresis, decreases levels of immunoglobulins more specifically and can process higher volumes of plasma. Kasperkiewicz et al. [79] reported the case of a 40-year-old patient with LABD who was responsive to yet experienced a recurrence of prednisone and dapsone [79]. Tryptophan-based adsorber IA (Immusorba TR-350, Asahi Kasei Corp., Tokyo, Japan) was then started along with the continuation of 7.5 mg/day of prednisolone and 1.5 mg/kg/day dapsone. The prednisolone was tapered and stopped after 11 weeks post-IA initiation. Dapsone was also tapered down over eight weeks. The patient remained in remission after 15 months. IA may be a treatment option in refractory cases or those with severe diseases requiring prompt remission induction. Some disadvantage to keep in mind about the tryptophan adsorber is the required coagulation testing daily due to its affinity to plasma particles such as fibrinogen- and bradykinin-degrading enzymes. Additionally, IA may not be available at all facilities, limiting its use.

Gluten-Free Diet

Linear IgA typically does not respond to a gluten-free diet (GFD) as there is no confirmed association between this and gluten-sensitive enteropathy (Celiac disease) [1]. One case report in which a patient had both lamina lucida LABD and gluten-sensitive enteropathy was treated with a gluten-restricted diet, which resolved jejunal abnormalities on biopsy as well as skin lesions [2]. Reintroducing a diet containing gluten caused the skin lesions and jejunal abnormalities to return. However, the patient did receive treatment with 50 to 100 mg of dapsone, which resolved her skin lesions before initiating a GFD. When the dapsone dose was lowered, the skin lesions reappeared; the subsequent combination of low-dose dapsone and GFD allowed remission of the disease. Even after discontinuation of dapsone, the patient remained lesion free on only a GFD. Therefore, a patient with underlying gluten-sensitive pathology who develops LABD may respond to a gluten-restricted diet, especially in combination with dapsone. Because diet restriction spares the use of medications, it may be attempted first, particularly in milder cases, to determine the response before initiating medication. However, a lack of evidence suggesting an association between LABD and a GFD may place undue stress on the patient due to the cost and lifestyle change that comes along with a GFD.

## Conclusions

LABD is a well-studied disease with many treatment options. Although dapsone and systemic corticosteroids have been established as first-line treatments, many patients are resistant to initial treatments and require other therapies. This paper summarizes and highlights the variety of options available in treating complex cases of LABD and provides an algorithm to guide the management of recalcitrant disease in both adult and pediatric populations.
